# Lesson Learned from Peer Volunteers in a Peer-Led Pain Management Program among Nursing Home Residents

**DOI:** 10.3390/ijerph16173097

**Published:** 2019-08-26

**Authors:** Mimi Mun Yee Tse, Shamay Sheung Mei Ng, Xue Bai, Paul Hong Lee, Raymond Lo, Daphne Sze Ki Cheung, Kin Cheung, Suey Shuk Yu Yeung

**Affiliations:** 1 School of Nursing, The Hong Kong Polytechnic University, Hong Kong; 2 Department of Rehabilitation Sciences, The Hong Kong Polytechnic University, Hong Kong; 3 Department of Applied Social Sciences, The Hong Kong Polytechnic University, Hong Kong; 4 Department of Geriatrics and Palliative Medicine, Shatin Hospital, Hospital Authority, Hong Kong; 5 Department of Human Movement Sciences, Vrije Universiteit Amsterdam, 1081 HV Amsterdam, The Netherlands

**Keywords:** volunteer, peer groups, pain management, nursing homes

## Abstract

Background: Chronic pain is common among older adults and is associated with adverse physical and psychological outcomes. Given the burden of pain and limited healthcare resources, devising innovative and cost-effective ways of managing chronic pain is of high priority. The aim of this paper is to explore the experiences and perceptions of peer volunteers (PVs) in a peer-led pain management program among nursing home residents in Hong Kong. Methods: Forty-six PVs were recruited and trained to lead a pain management program (PAP). The PAP consisted of one 1 hour session per week for 12 weeks. It included 20 min of physical exercises performed under the supervision of PVs, followed by 30 min of pain management education, including information on pain situations, the impacts of pain, the use of drugs and non-drug strategies for pain management, demonstrations, and return demonstrations of various non-drug pain management techniques. Quantitative data were collected from questionnaires (demographics, pain situation, and pain knowledge) for all PVs. Qualitative data (PVs’ experiences in leading the PAP, their perceived benefits, barriers encountered, and recommendations for improving the PAP) were collected at week 12 (upon completion of the PAP). Data were analyzed using the Statistical Package for Social Sciences and content analysis for qualitative data. Results: A total of 46 PVs were recruited (34 females, 74%), with a mean ± SD age of 61.0 ± 5.1 years. Thirty-one of them reported having chronic pain. Before the training, their self-rated pain knowledge was 40.0 ± 20.5 (maximum 100 points) while their actual pain knowledge score was 86.1 ± 10.6 (maximum 100 points). The PVs reported an improvement in their knowledge and skills after leading PAPs. No PVs reported having received any negative comments about their role in leading the PAP but mentioned that they had received feedback on how to improve the program. Conclusions: This study provides further evidence that peer-led pain management programs are feasible and can lead to positive experiences for the PVs. Peer support models are coming into wide use because they show promise in helping patients to manage chronic conditions. Peer volunteers will become important resources in elderly care. The barriers that were identified may lead to improvements in the design and planning of future PAPs.

## 1. Introduction

### Background

Chronic pain is common among older adults, with a prevalence of more than 50% among community-dwelling older adults [1] and 80% among nursing home residents [2]. It may be underreported as some older adults incorrectly believe that pain is a normal process of aging [3]. The consequences of chronic pain include impaired activities of daily living, mobility, depression and anxiety, and an increased burden on the healthcare system [2,4]. With populations continuing to age, prevalence rates for chronic pain are expected to increase. Given the expected burden and limited healthcare resources, an innovative and cost-effective method of managing chronic pain should be developed.

In this regard, peer support models [5,6] involving the provision of emotional, informational, and relationship support are being used to help patients manage their chronic conditions, with promising results [5,6]. A Cochrane review described positive outcomes in people with chronic conditions, including a reduction in pain, disability, and fatigue when self-management education programs were led by lay individuals rather than health professionals [7]. Peers are people of similar age and life experiences; thus, there is a higher level of rapport and less of a feeling of threat when one is supported by people like oneself as compared to professionals [7]. Peer-led programs may also cost less than those led by professionals [7,8].

Peer volunteers (PVs) are there to help patients manage their chronic conditions, including pain, with success and acceptance [5,6,7,8]. Indeed, the success of a peer-led program depends upon the feasibility of the PVs’ role [9]. Studies examining peer support have shown that PVs found their role to be satisfying, as they gained valuable new skills [10,11]. Therefore, it is important to understand why PVs volunteer, and what their expectations and experiences are in a peer-led program. 

The prevalence of pain among nursing home residents is as high as 70%–80% [12]. Nursing home residents are physically frail, live in “closed” nursing home environments, and may have difficulty seeking pain management strategies [2,3]. Indeed, older adults are often unwilling to report their pain to healthcare professionals, making the need to provide pain management education to nursing home residents a high priority [13]. To the best of our knowledge, there have been no previous studies on PVs’ experiences of volunteering in a peer-led pain management program among nursing home populations.

The aim of this paper is to fill this research gap by exploring the experiences and perceptions of PVs in a peer-led pain management program among nursing home residents in Hong Kong. It formed part of a larger research study, a clustered randomized controlled trial investigating the effectiveness of a peer-led pain management program in relieving chronic pain and enhancing pain self-efficacy among nursing home residents.

## 2. Methods

### 2.1. Study Design, Samples, and the Pain Management Program (PAP)

This study used a longitudinal design to examine quantitative and qualitative data provided by PVs who delivered a 12-week PAP to nursing home residents living in Hong Kong. Data were collected from questionnaires for all PVs at baseline (before attending the training) and at week 12 (upon completion of the PAP). 

The PAP started with 20 min of physical exercises performed under the supervision of PVs. This was followed by 30 min of pain management education, including information on pain situations, the impacts of pain, the use of drugs and non-drug strategies for pain management, and demonstrations and return demonstrations of various non-drug pain management techniques.

At the end of the session, the PVs helped the participants make portfolio entries on the activities of the day, to help them recall the various pain relief methods learned in each class.

### 2.2. Recruitment, Training of Peer Volunteers, and Fidelity Assessments 

PVs were recruited from the Institute of Active Aging (IAA) hosted by the Faculty of Health and Social Sciences of the Hong Kong Polytechnic University. They were mostly retired, highly educated people who were willing to volunteer their time to contribute to the community. They had largely been employed in professional and managerial positions. 

The criteria for being a PV were those: (i) aged 55 years or older; (ii) who had a score of >6 in the Abbreviated Mental Test to indicate that they had the mental/cognitive capacity to serve as older PVs; (iii) were able to attend training workshops and biweekly meetings with the research team for case reviews, discussions, and to reinforce strategies on pain management education; (iv) who had passed an exit test, including a knowledge test on pain management, a demonstration of various non-pharmacological practices, and an ability to use the teaching manual (the principal investigator (MMYY) and one of the co-investigators were the assessors, and supplementary classes were given to those PVs who did not pass the exit test); and (v) who expressed a willingness to lead the PAP in a nursing home. Fifty-eight individuals expressed interest in the study: 46 PVs attended the training workshops and completed the self-administered questionnaire, and 29 of them completed the training workshops.

The PVs attended four training workshops over a two-week period, and each workshop lasted for 2 h. Topics for the training workshops were: (i) discuss what a peer is; (ii) communication skills; (iii) client safety and confidentiality; (iv) managing crises and emergencies; (v) motivational strategies to enhance the compliance of the clients; (vi) demonstrations on the use of the teaching manual (i.e., “I can do it”); and (vii) various non-pharmacological practices. Training was conducted in small groups with the use of the following teaching methods: Dialectic lecturing (group), small group discussions, case sharing, demonstrations, and return-demonstrations (individual) on non-pharmacological pain management. The instructional model was group-based but the research team was also available for individual consultations. The return-demonstrations were designed as individualized coaching sessions to ensure that the skills were mastered 

With regard to the fidelity assessments, all PVs were observed three times (by random selection among the 12 sessions) when carrying out the PAP using a fidelity checklist. The fidelity checklist indicated the implementation of PAP in terms of four levels: Low/not observed; observed to a small degree; observed to a medium degree; and high implementation. The PVs demonstrated 90%–95% implementation at a high level in our present study, which indicated a high level of intervention fidelity. 

### 2.3. Data Collection

#### 2.3.1. Demographic Information

The questionnaire was completed by the PVs to obtain their demographic information, including data on their sex, age, marital status, educational level, occupation, medical history, and volunteer experience. 

#### 2.3.2. Pain Situation

The PVs were asked if they had any chronic pain. The intensity of their pain in the previous 24 h was assessed using the Chinese version of the Brief Pain Inventory [14] to determine the multidimensional nature of their pain, including its intensity and subsequent interference with life activities in the previous 24 h. The PVs were asked to rate their pain on a scale of 0 (no pain) to 10 (worst pain). This instrument is a reliable and valid measure of pain [14].

#### 2.3.3. Pain Knowledge

The PVs rated their pain knowledge before the training took place, and at week 12, upon completion of the nursing homework, using a 100-point Likert scale where a higher score indicated higher self-rated pain knowledge. Pain knowledge was assessed by having the PVs complete a pain knowledge questionnaire before the training took place and at week 12. The questionnaire consisted of 14 items about common myths on methods of managing (Appendix A). One point was given for each correct answer. A higher score (maximum 100 points) indicated a higher level of pain knowledge. 

#### 2.3.4. Qualitative Data

It was important to include both quantitative and qualitative data so that more feedback and comments could be collected from the PVs, in order to enhance the quality of the program. All PVs were invited to take part in an interview conducted by the research assistant. Field notes were taken during the focus group interview and were included in the analysis. The interview included open-ended questions in areas related to the PVs’ experiences in leading the PAP, their perceptions of the benefits, limitations, and barriers that they encountered, the usefulness of the PAP to the participants, and recommendations for improving the PAP. 

### 2.4. Data Analysis

Data were analyzed using the Statistical Package for Social Sciences (SPSS). Quantitative data were summarized using means (standard deviations) for continuous variables and proportions (n) for categorical variables. A paired sample t test was used to assess the difference in self-rated pain knowledge and pain knowledge score (two-tailed *p* < 0.05). 

For the qualitative part of the study, all PVs were invited to take part in an interview upon the completion of the PAP. They were questioned on their experiences in leading the PAP, their perceptions of the benefits and barriers that they had encountered, and on whether they had any suggestions on how to improve the program. The tape-recorded interviews were then transcribed and cross-checked by the research team to ensure consistency and accuracy. To achieve consistency and agreement on the meaning of the data, the research team compared, discussed, and agreed on codes, and then combined them with verbatim data to form categories/subcategories. Finally, a set of categories and subcategories with supporting verbatim data were generated to describe the experiences and perceptions of the PVs, as well as the barriers that they had encountered and their feedback on the content of the PAP. 

### 2.5. Ethical Considerations

Ethical approval was obtained from the Human Subjects Ethics Sub-committee of the Hong Kong Polytechnic University, and all participants gave their written informed consent prior to the collecting of data. Trial registration: ClincalTrials.gov (NCT03823495), 30 January 2019. 

## 3. Results

### 3.1. Characteristics of the Peer Volunteers

The PVs led the PAP in three nursing homes, for 60 nursing home participants who were suffering from chronic pain. They were 71–80 years of age, had lived in nursing homes for about 2 years, and were mentally sound with full awareness of time, people, and place. 

We made an announcement in the email system of the IAA regarding our PV recruitment and training sessions. Forty-six PVs responded to our invitation and joined the training session. Among them were 34 females (74%), with a mean ± SD age of 61.0 ± 5.1 years. Table 1 shows the characteristics of the PVs. The majority were married, possessed a university degree, and had a technical job. Almost all of the PVs had previous voluntary experience. Most of the PVs were invited by others to volunteer. Twelve of the PVs had chronic diseases, with hypertension being the most common.

### 3.2. Pain 

**Figure 1:**
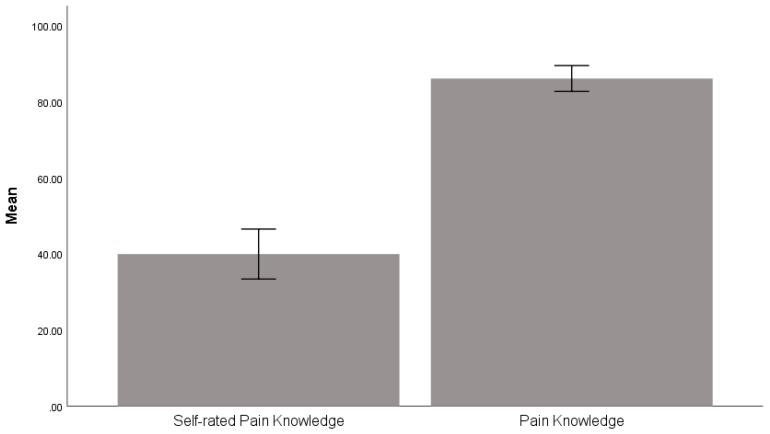
Comparison between the participants’ self-rated pain knowledge score and the actual pain knowledge score. *Note*. The error bar presents a 95% confidence interval.

Thirty-one PVs reported having chronic pain, with a mean ± SD pain score of 2.4 ± 2.0 out of 10. Before undergoing training, their self-rated pain knowledge was 40.0 ± 20.5. When their actual pain knowledge was assessed, a mean pain knowledge score of 86.1 ± 10.6 points was found. Questions that were incorrectly answered by most of the PVs included: “Pain is unavoidable and needs to be tolerated in the elderly”, “Visual stimulation does not have any effect on relieving pain”, and “Oral analgesics should be taken according to the severity of the chronic pain”. There was a significant difference between the self-rated pain knowledge and the pain knowledge score, with *t*(39) = 12.96 and *p* = 0.000 (see Figure 1).

### 3.3. Qualitative Data

Comments and feedback from PVs were organized as categories: Meaningful, helping themselves and helping others, boosted my self-worth, barriers encountered, and feedback on the content of the PAP. The data were arranged in a table format as in Table 2 below. 

## 4. Discussion

This study focuses on the use of peer volunteers in leading a pain management program for older adults suffering from pain. The prevalence of chronic pain is high, and chronic pain has an adverse impact on physical and psychological health. However, managing chronic pain in older adults is costly when funding and resources are inadequate and healthcare expenditures are increasing as the population ages. Therefore, the use of peer support would be an appealing strategy. 

Peer support models [5,6] are becoming widely used because they show promise in helping patients to manage chronic conditions. In this study, peer volunteers were trained to become important resources in elderly care. PVs reinforced the knowledge of the nursing home residents, re-demonstrated pain management strategies, praised the residents’ accomplishments, shared personal experiences and developed social bonds with them, and persuaded them to adhere to treatment recommendations. Furthermore, the benefits of using older volunteers are that they are not constrained by time and are a readily available resource. The cost and time required to train those PVs would be worthwhile if they can be empowered and continue to contribute to society by participating in the PAP.

To the best of our knowledge, this is the first study to explore the experiences and perceptions of PVs in a peer-led pain management program among nursing home residents. The findings of the present study can add to the body of knowledge on pain management. The outcome of this study should provide evidence of the effectiveness of a peer-led pain management program for nursing home residents with chronic pain. Consistent with the findings from other peer-led programs [15,16], the PVs in this study reported an improvement in their knowledge and skills. However, it was not feasible at this stage to analyze the changes in the self-rated pain knowledge score or actual pain knowledge score between the start of the study and at week 12. No PVs reported having received negative comments about their role in the PAP, although they mentioned having experienced barriers relating to communication, space, and privacy. These challenges need to be taken into consideration when planning and implementing future peer-led PAPs in nursing homes.

The PVs perceived that their role boosted their “sense of self-worth” which has been regarded as a powerful alleviator of stress and hopelessness [17]. A “sense of self-worth” also helps people to have a more positive interpretation of their own health [18] and to better cope with chronic diseases [19]. Future studies can explore the changes in physical and psychological health outcomes such as pain intensity, quality of life, and levels of happiness among PVs who led the pain management program. 

There are several limitations to this study. First, the findings relate specifically to peer-led PAP among nursing home residents in Hong Kong and may not be generalizable to other peer-led PAPs in other settings or other countries. Second, the PVs may have overemphasized the benefits of their participation, because of the time that they had spent and their emotional investment in the role. They might also have been concerned about making negative comments on their role. However, most of them were open about reporting the barriers that they had experienced. Third, the small sample size of peer volunteers in the present study constitutes a limitation, especially with regard to the qualitative data, and further studies on the experiences of PVs are needed. 

Nonetheless, the findings of this study are useful for future work on the implementation of peer-led PAPs. For example, the benefits reported by the PVs can be used to recruit PVs for future peer-led PAPs. 

## 5. Conclusions 

This study provides evidence that peer-led pain management programs are feasible and effective. The experiences and perceptions of PVs in a peer-led pain management program among nursing home residents in Hong Kong were positive. The perceived benefits of PVs included a self-reported increase in pain management knowledge and skills. The findings of the present study can add to the body of knowledge on pain management. 

## Figures and Tables

**Table 1:** Characteristics of the peer volunteers (*n* = 46).

Variables		*N* (%)	M	SD
**Sex**	Female	34 (74)		
Age, years			60.95	5.07
Age group	Under 60	22 (48)		
	60–70	22 (48)		
	70–80	2 (4)		
Marital status	Married	34 (74)		
	Single	4 (9)		
	Divorced	5 (11)		
	Widowed	3 (7)		
Level of education	Primary school	1 (2)		
	Secondary school	17 (37)		
	University	28 (61)		
Occupation	Physical laborer	2 (4)		
	Clerk	17 (38)		
	Technical job	23 (51)		
	Housewife	3 (7)		
Any chronic illnesses, yes		12 (26)		
	Diabetes	1 (2)		
	Hypertension	6 (13)		
	Heart disease	2 (4)		
	Cataract	2 (4)		
	Stroke	1 (2)		
	Arthritis	1 (2)		
	Cancer	2 (4)		
	Other chronic illness	1 (2)		
Chronic pain, yes		31 (67)		
	Head	6 (13)		
	Shoulders	8 (17)		
	Arms	9 (20)		
	Back	10 (22)		
	Legs	17 (37)		
Worst Pain score (Range: 0–9)			2.37	2.04
Previous voluntary experience, yes		40 (87)		
Invited by others to volunteer, yes		34 (74)		
Self-rated confidence in volunteering (100-point Likert scale)			78.7	16.3
Self-rated pain knowledge (100-point Likert scale)			40.0	20.5
Pain knowledge score			86.1	10.6

**Table 2:** Comments and feedback from PVs.

Categories	Comments and feedback from Peer Volunteers (PVs)
***PVs described leading the pain management program (PAP) as a meaningful experience***	I was appreciated by nursing home residents
	Nursing home residents were touched and said that they never expected us to be so nice to them
***Perceived benefits: helping themselves and helping others***	My pain is gone after volunteering in the program
	I feel happy by helping others
	I can see that the participants are happier and feel less lonely
	This program effectively relieves the pain of the participants
	My pain is gone after volunteering in the program
	I feel happy by helping others
	I can see that the participants are happier and feel less lonely
	This program effectively relieves the pain of the participants
***Boosted my sense of self-worth***	My family and friends recognized my achievement and were proud that I was a volunteer
	I get satisfaction in giving something back to the society and providing support to the participants
***Barriers encountered in leading the PAP***	Some nursing home residents had a hearing impairment, so that it was challenging to communicate effectively with them
	Some nursing home residents were too frail and required more assistance
	The space in the nursing home is limited, so we had to work things out with the nursing home in-charge
	We had to protect the privacy of each nursing home resident
***Feedback on the content of the PAP***	I like the PAP
	To improve the PAP, e.g., to remove the [section on] pharmacological management since it is not appropriate to teach nursing home residents about medications, which are kept and managed by the nursing staff
	The PVs focused on reminding residents to take the medications once given by the nursing staff and not to store up the medications

## Data Availability

The datasets used and/or analyzed during the current study are available from the corresponding author on reasonable request.

## Abbreviations

**IAA** Institute of Active Aging; **PV** peer volunteers; **PAP** Pain management program; **SPSS** Statistical Package for Social Sciences.

## Appendix A. Pain Knowledge Questionnaire

1. Chronic pain is defined as pain persisted more than three to six months.

Yes     No

2. Chronic pain can only be treated by medicine.

Yes     No

3. The effects of Panadol are anti-fever and killing pain.

Yes     No

4. Taking rest and reducing activity are the best methods to manage chronic pain.

Yes     No

5. Oral analgesic should be taken according to the severity of the chronic pain.

Yes     No

6. The time period of applying hot gel pad or cold gel pad on pain site is 1 h each time.

Yes     No

7. Music therapy helps pain sufferers to relax their body, mood and relieving pain.

Yes     No

8. Tasting tea is based on taste stimulation to relieve pain.

Yes     No

9. Pain killer can be categorized into morphine and non-opioid.

Yes     No

10. Visual stimulation does not have any effect in relieving pain.

Yes     No

11. Regular exercise can help to relieve pain.

Yes     No

12. The hot gel pad and cold gel pad need to be wrapped around with towel before applying on pain site.

Yes     No

13. Hot gel pad and cold gel pad can be used during sleep.

Yes     No

14. The massage technique is to use fingertips to press vigorously on pain site.

Yes     No
